# Evaluating and Engaging: Using Participatory Video With Kenyan Secondary School Students to Explore Engagement With Health Research

**DOI:** 10.3389/fpubh.2022.797290

**Published:** 2022-03-17

**Authors:** Alun Davies, Chris High, Nancy Mwangome, Rebecca Hanlin, Caroline Jones

**Affiliations:** ^1^The KEMRI-Wellcome Trust Research Programme, Kilifi, Kenya; ^2^Oxford Centre for Global Health Research, Oxford University, Oxford, United Kingdom; ^3^Peace and Development Studies, Linnaeus University, Växjö, Sweden; ^4^Trilateral Chair on Transformative Innovation, 4IR and Sustainable Development, University of Johannesburg, Johannesburg, South Africa

**Keywords:** schools, public, engagement, participatory, video, co-production

## Abstract

**Background:**

The growing ethical requirement to engage communities with health research has yielded diversification in approaches and targeted audiences. Conventional approaches like community “town-hall meetings,” laboratory open-days and focus group discussions, have evolved into new methods and audiences such as community drama and school engagement with health research (SEHR) involving learning interactions between researchers and school students. While engagement practices are diversifying, evaluations of these initiatives are rare in Low- and Middle-Income Countries (LMIC). This article focuses on the use of Participatory Video (PV) to explore the influence of the KEMRI-Wellcome Trust Research Programme's (KWTRP) School Engagement Programme (SEP) on the views and understandings of science and research among Kenyan state secondary school students.

**Methods:**

Twelve male and twelve female students from four coeducational schools were provided with film-making kits (1 per school), and a one-day PV training workshop. They prepared 22 short films over 8 weeks depicting their experiences and views of research and engagement and conveying their career aspirations. Schools were selected based on prior SEP participation; two schools having experienced different engagement approaches, and the others with no prior school engagement. Study data comprised footage and participant observation notes.

**Results:**

PV provided an opportunity to simultaneously engage and evaluate to inform practice. Through student-led filmmaking, PV stimulated conversations with students about research and engagement, enabling them to share their views in a way they felt was appropriate. These interactions offered an understanding of student gains from engagement, the depth of interaction required to address perceptions held about research and the potential unintended consequences of engagement. PV also provided insights into the context and complexity of life in which engagement is situated. Understanding this context is important because of its potential influence on participation in engagement activities. We draw on these insights to make two recommendations for school engagement practice. First is that PV can provide an enjoyable and insightful means of combining engagement with evaluation. Second, given that time for SEHR is competed for against other important curricular and extracurricular activities, SEHR practitioners must ensure that activities are as beneficial and enjoyable as possible to students.

## Introduction

As Public and Community Engagement to support health research is increasingly focusing on the need to inform research practice in low- and middle-income countries (LMICs), the range of approaches and goals have diversified (1–4). School engagement with health research (SEHR) is a growing field of engagement in LMICs which is not yet widely described in the literature (5, 6). At an international SEHR meeting held in Kilifi, Kenya in 2018, practitioners described four main categories of goals for facilitating SEHR (7). These were (a) raising awareness and stimulating dialogue about health research, (b) enhancing science education and nurturing student's interest in science generally, (c) strengthening capacity and nurturing the uptake of research careers by students and (d) promoting positive health behaviors. Given this broad range of goals, it is unsurprising that SEHR approaches are correspondingly diverse in terms of the types of activities they involve and the magnitude of their outreach. “Wide” engagement approaches, for example, day lab tours, online engagement with scientists and science magazine outreach/competitions (7) are likely to reach large audiences. Conversely, “deeper” approaches, including participatory approaches and Young Persons Advisory Groups (YPAGs) (7–11), are more likely to nurture longer-term relationships to facilitate co-learning and incorporating student views into research. Outreach in the latter however, is likely to be considerably smaller (12, 13).

Alongside the diversification in engagement approaches, there have been corresponding calls for appropriate evidence of engagement success (4, 14, 15). However, evaluating engagement is complex and challenging. First, because of the diversity in the ways in which the terms “community,” “public” and “engagement” are defined and interpreted (16–19). Second, engagement goals are numerous and sometimes in conflict with each other. For example, raising community awareness of the risks associated with research participation may be at odds with a goal of supporting recruitment (1). Third, challenges emerge in defining indicators to explore the extent to which engagement addresses intrinsic goals, such as trust, respect, and relationship building (20–22). Recruitment rates are argued to be inadequate indicators of the success of community engagement without a thorough understanding of participant's degree of voluntariness and understanding of the proposed research (1, 23). Lastly, the embeddedness of community engagement within health research institutes, with their dominant culture of experimental approaches (24, 25), is likely to influence the consideration of randomized control trials (RTC) for evaluation of engagement. However, while ethicists and funders increasingly describe engagement as critical for health research (2, 26), restricting engagement to only a proportion of a community to allow for a control arm might arguably be ethically challenging. Further, the complex non-linear nature of the engagement processes, and their need to be responsive and adaptable to constantly evolving and diverse contexts, makes the RCT approach practically challenging (27). As engagement approaches and goals continue to diversify, a corresponding broadening in approaches is needed to evaluate their impacts and influences.

Experimental and quasi-experimental approaches have been used to explore the impact of engagement between researchers and school children, ranging from post-intervention comparisons of participant to non-participant responses and attitude/knowledge questions or Likert items (28–31), to pre-post designs and cluster randomized control trials (6, 32). Qualitative methods such as in-depth interviews (IDI) and focus group discussions (FGD), are commonly used in the evaluation of SEHR activities, mainly to explain quantitative findings, but also to gain deeper insights into the influence of engagement and to describe the process (33–39). A few studies have drawn on more novel approaches to explore the effects of various SEHR approaches. For example, comparisons of the questions students have asked researchers before and after interaction (40) or exploring the impact of interactions on the way in which students depict scientists in their drawings (41). While documented evaluations of SEHR in high income countries, for example, USA, UK and Australia are common (33–39), documented research on the impact and influence of engagement between health research and schools in sub-Saharan Africa is very rare (6). The studies described (28–41) focus mainly on the impact and influence of the activities on student attitudes and views, providing only sparse descriptions of the context in which SEHR takes place and how this might influence outcomes for participating students.

Further, it could be argued that the rigidity of surveys, and challenges with facilitating meaningful participation of children in qualitative methods such as FGDs led by researchers (42), may only offer limited opportunities for students to engage with researchers and contribute to steering the conversation.

The use of participatory methods to evaluate SEHR approaches has not been described in the literature. However, participatory methods have long been used in the field of development (43) and a participatory visual method which is currently gaining popularity and use in community engagement for both “development” and “research” is PV (44). PV is a method which has been used to open up spaces for discussion and enable participants to create their own films to voice their concerns and take action in determining their own development (45). It has been used in health promotion (46–50), to evaluate community development projects and programmes (51–53) and other areas such as engaging participants with climate change (54) and neighborhood planning (55). Lemaire and Lunch (51) argue that “outsider”-based evaluations conducted by external evaluators have the potential to be extractive and disempowering. They postulate that PV can mitigate the risks of external evaluation and better reflect the priorities of project beneficiaries by allowing project participants, described as “insiders,” to participate in evaluations. While “practical participatory evaluation,” directly involving community members and project staff, may enable appraisal of project outcomes (56), using PV is argued to augment evaluation through incorporating a transformative dimension (51). The use of PV in the co-production of knowledge related to participant's experience of a project has the capacity to facilitate communication between several groups through the video outputs whilst enabling the evaluation of project influence (51). “Knowledge co-production” has been recently defined as an “Iterative and collaborative processes involving diverse types of expertise, knowledge and actors to produce context-specific knowledge and pathways toward a sustainable future” (57). Within the context of SEHR, PV can offer an opportunity for students to collaborate with researchers on the co-production of knowledge relating to their experience of engagement and research and its impact on their lives. A co-production process can strengthen relationships between researchers and participants and generate reciprocal and mutual benefits (58). Participatory arts-based approaches such as PV may be particularly suited for evaluating SEHR because they can enable participants to interrogate and question research practices, generating counter-narratives and co-produced knowledge in a way that can transform engagement practice (59).

Participatory visual methods are increasingly being used in research with children and young people (60, 61) in a range of contexts including advocating for climate change adaptation (54), exploring issues facing disadvantaged youth (62, 63), and engaging school children with STEM (Science Technology Engineering and Mathematics) to facilitate deeper learning of scientific concepts (64). PV has been described as a method which respects children as being knowledgeable (62). When carefully facilitated, PV has the capacity to challenge power hierarchies between researchers and study participants (65). This is arguably of particular importance for research involving children because, in addition to social, cultural, ethnic, educational and wealth differences between researchers and participants, age differences could heighten the potential power dichotomy, inhibiting open discussion. In view of this, Thomas and O'Kane (66) present the case that participatory research is particularly suited for research with children because it can address power differentials both through transferring more control of the research to children and making use of enjoyable procedures which align themselves to the way in which children see the world. However, Gallacher and Gallagher (60), though supportive of participatory methods, question their capacity to be universally democratic, emancipatory, and empowering for children. They caution that a pedagogic embodiment of adult researchers “empowering powerless children,” could result in children conforming to adult agendas and being disempowered in the process. Existing power dynamics within the participant group need also to be carefully and sensitively managed to ensure that the participatory processes don't reinforce them (62). Like other methodological approaches such as surveys, PV is not without challenges, and like other qualitative approaches, it requires constant reflexivity and awareness of the potential influence of power imbalances on the insights and experiences shared (54, 60, 67).

Given the value of PV outlined above, and acknowledging the power dynamics raised, this paper describes the process and outputs of a PV approach to evaluate a SEP in coastal rural Kenya with the aim of understanding the potential for the use of PV in SEHR. It provides a description and exploration of PV as a method for evaluating SEHR, offering insights into how its use provided understanding of the contextualization of SEHR activities within the lives of students.

## Methods

### Study Site

This study was conducted in Kilifi County, on the Kenyan coast, the location of the KEMRI-Wellcome Trust Research Programme (KWTRP). The KWTRP, established in 1989, employs over 800 people and conducts epidemiological, social, laboratory and clinical research aimed at improving health in the region. The KWTRP has a public and community engagement strategy, first established in 2005, which provides a broad range of fora where researchers and the public can engage and learn from each other. One component of the strategy is the SEP which facilitates engagement between researchers from the KWTRP and more than 4,000 students from over 50 Kenyan public primary and secondary schools every year. The SEP was initiated in 2008 to draw from KWTRP's human and lab resources toward contributing to local school science education in a context where public secondary schools are characterized by large class sizes, poorly resourced laboratories (68, 69), and according to local teachers, limited opportunities to learn about science. SEP activities have several aims. These comprise stimulating an interest in science and research related careers, raising awareness of locally conducted health research and promoting positive attitudes toward health research (5).

In 2014, a study funded by Wellcome was established to evaluate the outcomes of various forms of SEHR as implemented by the SEP (6). Forty secondary schools in Kilifi were involved in the KWTRP SEP at the time, and the programme and its development are described in more detail elsewhere (5). To summarize, in collaboration with school principals and the county director of education, 10 schools were invited on an annual rotational basis to participate in “face-to-face” (FTF) SEHR activities. These included student lab tours, interactive discussions with research staff about their work, online interactive discussions about science with researchers through a platform called “I'm a Scientist, Get me out of here!” (IAS) (70), researcher visits to schools to give career talks and inter-school science. The remaining 30 secondary schools were invited to participate in “less intensive” (LI) engagement activities comprising online engagement and inter-school science competitions only.

**Figure 1:**
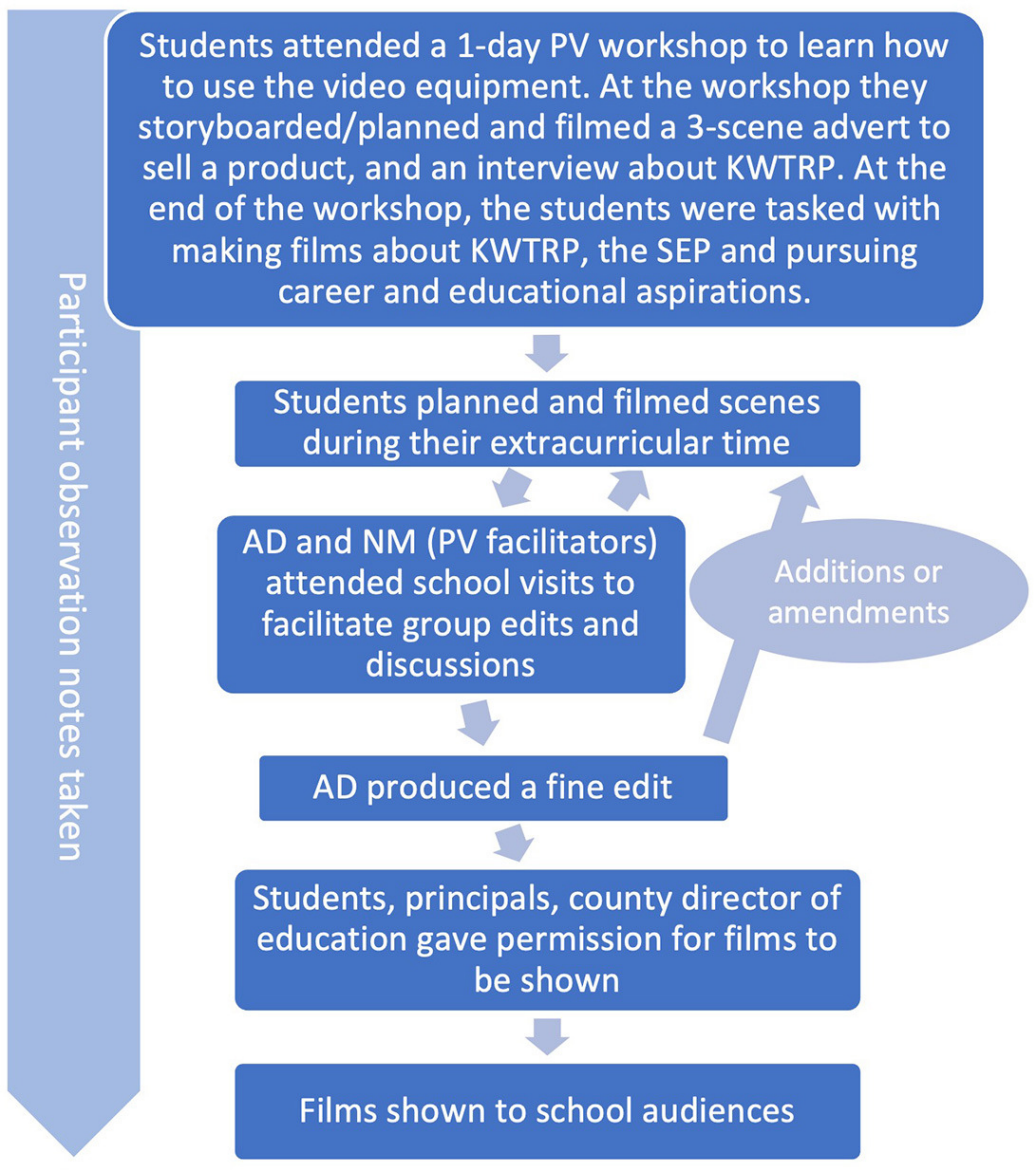
The PV process.

Between 2014 and 2016, the SEP activities described above were evaluated using a mixed methods approach summarized in Table 1. The purpose of the evaluation was to understand the impact and influence of engagement on: (i) students' interest in science and career aspirations; (ii) awareness of locally conducted health research; and (iii) attitudes toward health research (6). The evaluation was conducted among five FTF schools, five LI schools, and five control schools (C). The five control schools had not previously participated in SEHR, but were scheduled to be incorporated into the SEP after the evaluation was complete. Schools were purposively assigned to arms A, B and C to maximize the similarity between the 3 arms in terms of size of school (numbers of students), boarding/day, IT resources, and performance in external examinations. The mixed methods design, summarized in Table 1, is discussed elsewhere (6), and comprised three components. The first was a pre- and post-engagement student survey, and the second was a qualitative component involving interviews and focus group discussions with students, teachers, researchers, parents and community leaders. The third component, and main focus of this article, was a PV component with 24 students (outlined in Figure 1: The PV process).

**Table 1:** Mixed methods evaluation design.

	**Arm 1: Face-to-face engagement (5 schools)**	**Arm 2: Less intensive engagement (5 schools)**	**Arm 3: Pre-engagement (5 schools)**
Feb–Mar 2014	• Pre-engagement student survey (*n* = 491) across 15 schools		
May–Nov 2014	*Face-to-face activities: Lab tours; researcher visits to school; participation in inter-school science quiz; and “I'm a Scientist”–online platform*	*Less intensive activities: participation in inter-school science quiz; and “I'm a Scientist”–online platform*	*No engagement activities until 2016*
	• Teacher IDIs and Student FGDs	• Teacher IDIs and Student FGDs	
Nov 2014–Feb 2015	• FGDs and IDIs with students, teachers, community leaders, education stakeholders, participating KWTRP staff • Post engagement student survey (*n* = 491) across 15 schools		
Feb–July 2015	• PV with 1 school	• PV with 1 school	• PV with 2 schools

### Objectives of the PV Component

Drawing from an ethnographic perspective, we felt that combining PV with participant observation could enable us to draw inferences on SEHR based on observations and discussions of students working on a project over an extended period of time (71). The primary purpose of adding a participatory visual method to the overall mixed methods design was to explore the influence of different forms of the SEP (FTF and LI) on students' understanding of and attitudes toward the KWTRP and health research, and on their career aspirations. Specifically, we used PV to explore the following research questions:

a) What were the students' experience of SEP and how did it influence their views about science and their career aspirations?
b) What is the SEP's influence on student's understanding of and attitudes toward KWTRP and health research?
c) How could a PV process nurture further engagement with KWTRP and SEP?

### Procedures

As shown in Table 1, the PV component was the last in the sequence of evaluation data collection activities, affording the ability for the purposive sampling of four schools to represent the range of experiences and participation in the SEP activities, and the SEP evaluation. FTF school 1 (FTF1) and LI school 1 (LI1) were selected based on their full participation in FTF and LI activities, respectively, and hence their capacity to share views on all aspects of the SEP. Two control (C1 and C5) schools were selected to explore whether student understanding, attitudes and aspirations differed to those of students who had previously engaged with health research. We purposively selected C1 and C5, schools with high and low survey participation rates, respectively, to yield a range of views in terms of prevailing attitudes toward KWTRP in the schools.

Groups of six students, three male and three female, from each of the four schools were invited to take part in the PV project spanning the second school term between the 4 May and 31st July 2015. A group size of six was selected to enable two students to operate the camera and microphone whilst allowing the remaining 4 to participate in interviews or small plays. In each of the four schools, form 2–3 students, aged between 16 and 18 were selected purposively, through consultation with the principal, to represent a range of participation in SEP activities (for FTF and LI), a gender balance and students who the principal felt would be able to share their views confidently.

The PV process, comprising an initial workshop and several follow-up sessions in described in Figure 1. Two initial one-day PV training workshops were held at the KWTRP; one for schools FTF1 and LI1 and the second for schools C1 and C2. The objectives of the workshops were to (a) create a rapport between AD, NM and the students, (b) familiarize the students with the equipment and techniques, (c) get the students started in making storyboards (a sequence plan of film scenes) and short films, and (d) to have fun (45). At the workshop students learned how to assemble and use the kit, how to storyboard and film an interview, and about group-editing. To facilitate this learning the students were tasked with storyboarding and shooting three-scene television adverts to sell a product of their choice. During “group editing,” AD, NM and the students reviewed the footage on the laptop editing suite, and the students decided which scenes to be included, omitted and trimmed, and the order of scenes. At the end of the workshops, each group of students were provided with a camcorder to take back to their schools, which the schools eventually retained. The groups were tasked with planning and making several 5-min films in their extracurricular club-time. Given that the primary purpose of the PV was for evaluating the SEP, the students were asked to make films about their experiences of KWTRP or SEP and about pursuing career and educational aspirations (and what might influence this). Beyond this, no restrictions were placed on the content, number, or the type of films made. AD and NM are fluent in Kiswahili and English and students were given a free choice of which language to use for their videos. Students were guided on taking care to only film people if they gave consent for being filmed.

Four follow-up sessions were undertaken at each school fortnightly, involving NM and AD and the six students during “extracurricular club-time.” The first three follow-up sessions comprised reviewing, discussing and group-editing of filmed footage. Discussions often led to film modification, which involved an iterative process of re-filming and subsequent group edits. This led to “co-production” of films and knowledge. Each of these sessions lasted between 40 and 90 min depending on the time available during the after-lesson period. In the fourth follow-up session, the films were shown to the school principal and then to school audiences. Observation notes were taken throughout the sessions.

During group editing sessions, student suggestions were noted and later addressed during the “fine edit.” Because fine editing is costly in terms of time (46, 72), this was done by AD at KWTRP. This entailed adding scene transitions, titles, sub-titles, name tags, sound effects and soundtracks, based on the students' suggestions. The core content of the films was not altered in the fine editing process. Draft film projects were exported to MP4 media files to show students. The students were free to make alterations, either through re-shooting or making suggestions for further edits, until they were happy to give overall approval for the final film draft.

Within their groups, students decided which audiences to share the videos with. Schools FTF1, LI1 and C1 opted to show the films to their entire form 2 year groups and a separate showing for their teachers, while C2 wanted to show the films to the entire school. All films were reviewed and approved for showing by school principals and the county education officer.

### PV Data Collection

The PV process generated two sources of data:

- Participant observation notes from AD and NM collected over all sessions. These were hand-written notes, providing detailed observations of story-boarding, group-dynamics, discussions, decision-making and direct quotes; and
- The edited media produced during the workshops and follow-up sessions.

Following interactions with students, NM and AD had de-brief discussions to reflect on student experiences and session discussions and add to observation notes. Observation notes were also taken during and after the video showing sessions with individuals and audiences. All notes were typed, and PV media were transcribed and translated from Kiswahili to English. All transcripts and notes were entered for coding into NVivo 11.

### Data Analysis

A thematic framework approach was used to analyze the data (73, 74). This involved familiarization with the data through repeated reading and re-reading of the observation notes and film transcripts, generating codes, and sorting them into overarching themes. The codes were then placed in matrix charts, which enabled a comparison of student insights, views and experiences across the FTF, LI and C groups. The framework approach allowed flexibility in exploring hypothesized, as well as unintended or unplanned, influences and outcomes of SEP. A combination of inductive and deductive approaches were used in the analysis, generating four overarching themes. The first three themes were predetermined at the outset and focus on the evaluative data generated by PV about the SEP activities. They respond directly to the research questions specified in the section describing the Objectives of the PV Component. In the fourth emerging theme, we explore how PV provided valuable insights into the context in which SEHR is situated. Understanding this context is important because of its influence on participation in, and commitment to, the engagement process.

### Addressing Potential Ethical Concerns

**Figure 2:**
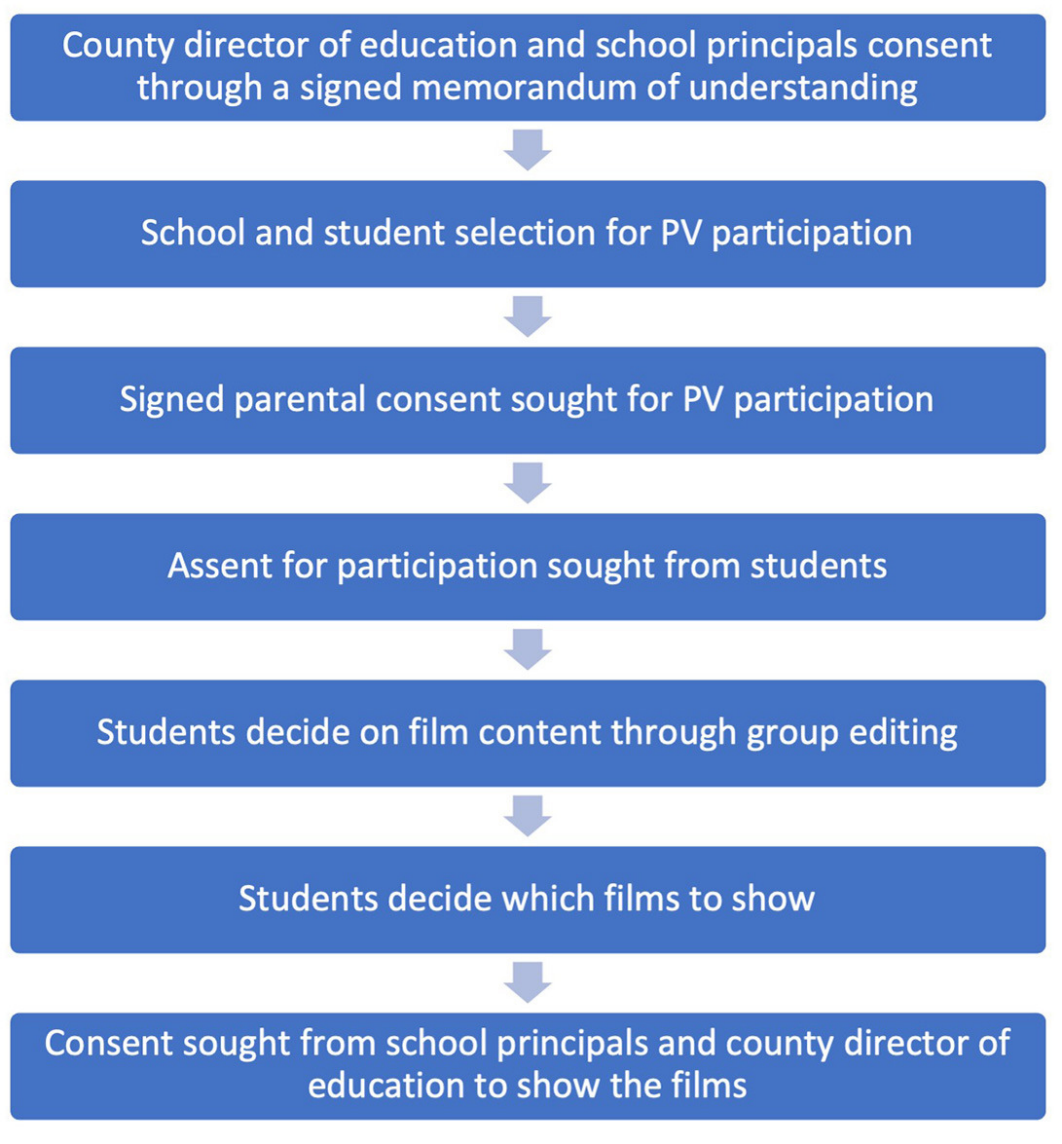
Multi-staged consent/assent process.

Ethical challenges in this study have been described elsewhere (44). Of specific concern was a potential risk that sharing personal information could lead to participating students being stigmatized. Two strategies were used to address this. Firstly, a multi-staged consent procedure (44, 75) was used in an attempt to ensure that students, parents and teachers and the county education officer were able to consent or withdraw throughout the filming process and the media sharing. This multi-staged consent/assent process is summarized in Figure 2. Secondly, the group-editing process enabled students to directly control the content of the films. Once the films were prepared, permission to show the films to different audiences was sought firstly from the participating students, secondly from the school principal, and lastly from the Kilifi Education Office. Students and principals provided signed approval of the films selected for showing to wider audiences. Participant's wishes to not show, or re-edit films were respected and acted upon.

Ethics approval was granted by the Kenya Medical Research Institute Scientific Ethics Review Unit: SSC 2672 - “Evaluation of the scaling up of the KEMRI-CGMR-C's School Engagement Programme in Kilifi.”

## Results

### Video Outputs

Over the 8-week period of the PV process, the students made a total of 22 videos. The videos, their presentation style, who participated in making them and key summary observations are shown in Table 2. The films were shown to audiences of students and teachers in the school, and this universally nurtured a great deal of excitement. In this article however, we focus on data generated through the media production and the observation of the student participants.

**Table 2:** Summary of the films produced by students.

**Task**		**Style and participants**	**Key summary observations**
Learning exercise: Make an advert to sell a product	FTF1 vid1	Commercial TV advert aimed at selling a notepad	Students followed the instructions and created a simple TV style advert.
	LI1 vid1	Short 30 second advert promoting the value of education	Students perceived a need to promote education in the community.
	C1 vid1	Short 30 second advert promoting HIV services and voluntary HIV counseling and testing	Students perceived a need to promote education about HIV.
	C2 vid1	Short 30 second promotion of the student's school and it's attributes	Students perceived a need to promote education in the community.
	C2 vid2	Musical ‘rap’ within the group depicting the value of their school	Students expressing pride in their school.
Task 1: Make films about your experiences of KWTRP	FTF1 vid2	Interviews within the group about KWTRP and SEP	Describes negative impact of Malaria and the benefits of research. Provides evidence that students have learned about KWTRP through SEP.
	FTF1 vid3	Role play-KWTRP researcher giving a career talk followed by a group poem	Evidence of SEP impact on students: role-play references culturing microbes and other SEP activities referenced. Scientists depicted as inspiring and motivating for students
	LI1 vid2	Interviews within the group about KWTRP	Range of community descriptions of KWTRP depicted: “benefit to society;” ‘the community do not know;” and “others think badly” of KWTRP. Descriptions of KWTRP as: health providers (lifesavers); educating children; hospital builders; and an AID organization treating people for free.
	LI1 vid3	Interviews about KWTRP	KWTRP perceived as a health provider. SEP activities enjoyed by students but had an unintended consequence of jealousy among non-participants.
	LI1 vid4	Play depicting KWTRP going around the community giving opportunities for people to be trained as health researchers. One decliner suffers the consequence of future joblessness	KWTRP depicted as benevolent-building hospitals; training youth and paying medical bills. Issues raised: limited understanding of qualifications required for KWTRP employment; lack of school fees; joblessness; power relations; peer pressure and lack of belief in education dissuading students from education.
	C1 vid2	Interviews within the group about their understanding of KWTRP	Students were uncomfortable in answering questions about KWTRP and displayed a range of understanding/value of KWTRP: ‘they come up with medicines to help cure sick in society and reduce mortality’; ‘they provide jobs for locals; limited understanding of requirements for KWTRP employment.
	C1 vid4	Documentary comprising interviews with students, teachers and KWTRP staff aimed at addressing student questions about research and KWTRP	With the exception of one participant (‘conducting research on medicines to save lives’), there was limited understanding of the role of KWTRP's census and blood drawing. Range of opinions about KWTRP: good organization; using people like guinea pigs for research; and devil-worshippers.
	C2 vid3	Interviews exploring community views about KWTRP and research	Range of community interpretations and attitudes expressed: KWTRP addressing disease and epidemics; KWTRP as a health provider; creating jobs; believed to be ‘devil-worshippers’ (related to blood-sampling for research).
	C2 vid5	Interviews with students (outside the group) teachers and the school cook, exploring community views about KWTRP and research	Range of interpretations, attitudes and beliefs: KWTRP described primarily a health provider and so have benefitted people; some described KWTRP as people doing research to reduce mortality; blood samples taken for unknown use–possibly devil-worship; students can benefit educationally from KWTRP.
Task 2: Make films about your educational and career aspirations (and what might influence this)	FTF1 vid4	Play about sexual coercion, peer pressure, pregnancy and school drop-out	A delinquent boy approaches a girl and asks her to arrange a sexual liaison with her friend for money. The girl makes the arrangement (pocketing half of the money) and her friend becomes pregnant and drops out of school.
	FTF1 vid5	Interviews followed by a role play about career aspirations	Students depict receiving careers inspiration from: family members, KWTRP SEP; the need to address HIV; and a perceived lack of doctors. Researchers described as positive contributors to community health. Students demonstrate a good understanding of KWTRP. Financial barriers to pursuit of education acting against aspirations.
	LI1 vid5	Play within the group with one additional member from outside the group, about poverty education and early marriage	In a poor family, the jobless father decides, against the mother and daughter's will, that the solution to the family's financial problems is to take the daughter out of school and marry her off for dowry. A teacher persuades the father to keep the daughter in school. Societal pressure for early marriage of girls.
	FTF1LI1 (together) vid1	Play students from FTF1 and LI1	Girls receiving unwanted sexual advances from boys on the way to school.
	C1 vid5	Students' information film about their school	Students expressing pride in their school, and highlight: the long distance of the school from nearest town; and resource challenges faced by rural schools.
	C2 vid6	Play within the group expressing students' dissatisfaction with corrupt employment practices	The play highlights barriers to employment: bribery for scarce jobs; the power of employers and wealthy people who can afford to bribe.
	C2 vid7	Role play	Lawyer describes her struggles to achieve career progression through challenging circumstances: single parenting; lack of tuition fees; and long distances to school (specific vulnerabilities for girls implied).
	C2 vid8	Play about the impact of drugs on education	Students tempted by an outsider to take drugs on the way to school, supported by peer pressure. They return to class intoxicated and cause a riot. They are persuaded by the school head that drugs are harmful.

### What Was the Student's Experience of SEP and How Did It Influence Their Views About Science and Career Aspirations?

With varying degrees of engagement, students across all groups were aware of the SEP and articulated their understanding of its roles in their films. Despite having no exposure to SEP activities, students in control schools were also aware of the programme.

*KEMRI is making these sciences to be upheld positively by the students who really are learning in various secondary schools in Kenya. (Male, School C2 vid 5)*.

FTF1 students, who had received the face-to-face engagement package, both in their films and group discussions, articulated a greater depth of understanding the SEPs goals:

*KWTRP is engaged in [the] school programme by introducing the young generation, the upcoming youth to know what KWTRP is and what it does to the community. It also engages in school activities like providing symposiums, science fairs, and also for the students who have finished their form 4 course, they are being trained on how to come up with best careers in life [through an] attachment for a period of not less than 3 months. (Male, School FTF1 vid 2)*.

Of the 11 films made by students from the two intervention schools (FTF1 and LI1), six films referenced experiences of the SEP, described some of the intervention activities and shared their feelings about them (Table 2).

“*Yea, it was interesting because as for me, it was my first time to talk to scientists, so I found it quite good.” (Male, School LI1 vid2)*.

Students from these schools, through their discussions and in their interviews, described SEP activities as being “*fun,” “enjoyable,”* and “*motivating.”* In a poem created as part of the PV exercise, students from school FTF1 described specific SEP interactions with researchers influencing their awareness of science related careers, motivation in science subjects and awareness of research:

“*When I see and interact with scientists, I feel motivated.” (Male, School FTF1 poem vid 3)*.

“*As I have interacted with KWTRP in many activities, I have felt motivated, and I have improved in my science subjects.” (Female, School FTF1 vid 3)*.

LI1 students, in their films and review discussions, placed more emphasis on the novelty of meeting with scientists, and the benefits of learning about communication through the internet. In comparison, the FTFI students focused more on the influence of the SEP on their attitudes to science.

*P1: that activity was so [much] fun. To most of us [we] didn't know how to use a laptop, we were taught how to use them, to chat with people from different places in Kenya… We are so grateful to KWTRP and we wish them all the best and to continue with more activities to encourage students on those scientific subjects to develop more careers. (Male, School LI1 vid 3)*.

These findings might be explained by the opportunities afforded by the LI activities for extended use of the internet and interactions with scientists, and the additional activities experienced by the FTF1 students.

Novel engagement approaches like IAS (and similarly the PV), appealed to FTF1 and LI1 students and offered opportunities for communication and interaction with a range of people using media which was new to them. It is important to note that the majority of comments made by students about SEP were very positive with very few criticisms. This suggests that SEP provided opportunities for students, the first opportunity for some, to interact with researchers in a way that the students reported as being enjoyable and beneficial.

Students from all four schools described a variety of desired careers in their films. FTF1, LI1 and C2 expressed a desire for medicine-related careers. In contrast to schools LI1, C1 and C2, students from school FTF1 described a desire for a repertoire of careers similar to those specifically encountered through the SEP activities, in some cases, referring directly to specific research staff they encountered:

“*My visit to KWTRP laboratories to see microorganisms being cultured has inspired me to become a microbiologist.” (Female, School FTF1 vid3.)*.

“*I remember the nurse who talked about human resource management.” (Female, School FTF1 vid5)*.

Other examples of inspiration described by School FTF1 students, and likely to be related to SEP encounters, were a desire to attend campus, achieve a PhD, become a nurse, study anatomy and be a “*researcher the community can be proud of”* (Male, School FTF1 vid3). The wider range of desired careers related to those encountered at KWTRP and described by FTF1 students, provide some evidence that engagement broadened students' ideas of what they might aspire to or, in other words, their “*repertoires of possible future selves*” (76). Comparison of pre and post engagement student surveys, described elsewhere (6), also yielded evidence that FTF engagement, to a greater extent than LI, promoted positive attitudes toward science, scientists and research-related careers.

### What Is the SEP's Influence on Students' Understanding of and Attitudes Toward KWTRP and Health Research?

To explore student understanding of KWTRP across all groups, and the influence of SEP on FTF1 and LI1 students, they were tasked with preparing for and filming group interviews responding to their own questions about KWTRP. Across all groups questions were similar, for example, “Describe the work of KWTRP?” and “What is health research?” Acknowledging that students across Kilifi County learn about KWTRP from a range of sources, NM and AD observed differences across the groups in terms of student confidence in articulating the work of KWTRP. Predictably, students with more exposure to researchers through SEP, specifically FTF1 students, were generally able to describe the work of KWTRP more accurately and with more confidence than the other groups.

*P1: KEMRI is Kenya Medical Research Institute. KEMRI do research of different diseases such as malaria and pneumonia. They have come up with means and ways of preventing and curing them for the benefit of Kilifi residents. (Male, School FTF1 vid2)*.

Compared to FTF1 students, the C2 group side-lined questions requiring their own understanding of the KWTRP, opting instead to describe community views about KWTRP. In contrast, C1 students more openly expressed their difficulty in responding to the knowledge questions about KWTRP which they themselves had set. This resulted in an observable temporary lapse of confidence and frustration among group members. In a follow-up discussion, the students acknowledged that they found the activity challenging with one student summarizing that “*It's because we don't know about KWTRP” (C1observation notes)*.

Student films included a variety of interpretations of the roles of KWTRP. However, the ambiguity demonstrated in the LI1 and C films was less apparent in the draft films made by the FTF1 students. The interpretations of the role of the KWTRP in the LI1 and C school films included descriptions of KWTRP as a healthcare provider (LI1, C1 and C2), in facilitating blood donation/transfusion services (School C1), in conducting individual diagnostic tests, and as educating community members and school students (LI1 C1). The quote below highlights a common therapeutic misconception of research, and how a diagnostic test done as routine care at a hospital where research is also conducted, is interpreted as a medical research procedure.

“*My baby breathed so fast that I became worried that she might die! But they have done a good research on her and now they are giving her drugs and she is better.” (Female, School C2 vid3)*.

Given KWTRP's history of equipping and furnishing rural clinics in preparation for clinical trials, treating research participants, engaging with school students, and drawing blood samples for research, it is not surprising that the main roles of KWTRP may have been misinterpreted by students.

A diverse range of attitudes about the KWTRP were expressed across the groups. Positive attitudes relating to benefits community members felt they received from KWTRP were frequently depicted and expressed in the videos from all participating groups. These benefits included a perceived contribution to individual and community health through direct health care provision, provision of transport to hospitals and clinics, building health clinics in the community, research processes leading to reduced mortality, and KWTRP's contribution to employment opportunities in that area.

“*It has helped the community in research of outbreaks of diseases, yeah, it has done research on diseases and KEMRI has been able to come out with solutions.” (Male, School LI1, vid 2)*.

“*KWTRP is all right. And those people who despise it, you know, Swahili people say “you only praise the rain if you've been rained on.” Now, the one who hates it is the one that hasn't encountered a problem to go and benefit from there. (Female, School C2 vid 5).”*

The last quote voices an opinion that negative beliefs about KWTRP were a consequence of community members not feeling direct benefits from research or KWTRP. C1 and C2 students described beliefs within the community that KWTRP's work was associated with devil worship. In both cases this was expressed as beliefs among “*some people”* within the community, as opposed to the participants themselves. Students attributed this perceived association with a community suspicion of the need for KWTRP to draw blood from research participants (C2 vid3), or due lack of community understanding of the roles of KWTRP. Student's explanations for the sources of rumors: “*It's because we don't know about KWTRP”;* and linking blood drawing to devil-worship, is consistent with the notion proposed by Marsh et al. (77) of “half-knowing” leading to rumor. Interestingly, negative beliefs about the KWTRP were restricted to the films made by the groups from the C1 and C2 schools. This might suggest that the SEP had produced a positive influence on student attitudes toward the KWTRP in the LI1 and FTF1 schools. This was corroborated by the quantitative and qualitative components of the evaluation described elsewhere (6).

### How the PV Process Nurtured Further Engagement With KWTRP and SEP

The PV process and follow-up visits offered opportunities, over 6 weeks, to gradually create a conducive rapport between the AD, NM and the students. This facilitated mutual-learning and knowledge co-production. During the initial workshop, anxiety and a lack of confidence, specifically among FTF1 and LI1 girls and the C1 students, were observed through outward expressions of shyness and reluctance to communicate. C1 students were frustrated at being unable to respond to their own knowledge-based questions about KWTRP, and FTF1 and LI1 girls remained quiet during group discussions.

Evidence of shyness among the girls comprised observations of the lowering of their eyes, hiding their faces when films were shown, and remaining very quiet during follow-up discussions. This, to some extent, enabled the boys to dominate the discussion during the first stage of the process. Interestingly, this shyness was not apparent in the films they made but materialized only during group discussions and film showing sessions. Among students from schools FTF1 and C2, both boys and girls expressed enjoyment throughout the process while some of the LI1 and C1 girls expressed periodic shyness. Observation notes describe most students overcoming their shyness over the first couple of sessions.

In response to this initial reticence, AD and NM employed several strategies to make workshops and follow-up visits informal and enjoyable. These comprised, (a) encouraging the students to play with the equipment with minimal facilitator intervention, (b) making students “swap roles” to nurture the participation of less dominant group members, (c) encouraging the students speak in the language they felt most comfortable with. Students were enabled and encouraged to practice and repeat scenes as much as possible and AD and NM made a conscious effort to praise all aspects of their participation.

Over the duration of the PV component, relations between the schools and AD and NM were strengthened and this was evident in various ways. A growth in outward displays of student enjoyment and confidence were observed over the duration of the project, evidenced by increased tendency to smile, laugh and request for repeat showing of films. The warmth in which students and teachers welcomed AD and NM to follow-up visits also increased over the project. This was most marked in control group C2 where big handshakes and youth greetings encountered in some of the student dramas were frequently used by both researchers and students: “*Vipi masela? Mambo shega!”* (Hi guys, things are cool!) (C2 visit3). Further evidence of an increasing confidence and assuming control of their films, across all groups comprised: requesting the equipment be available beyond originally agreed times (extracurricular club time and lunchtime) for independent filming (FTF1 Vids 4&5; LI1 vid 5; C1 vid5; and C2 vids 5,6,7&8); reviewing material independently, and modifying scenes/content/articulation and/or deleting scenes they felt should be omitted (FTF1 vid 3, C1 vid 5, C2 vids 5,6,7&8); active participation in critiquing, editing, and modifying films (all groups throughout); being very definite about which films could or could not be shared with an audience (FTF1, LI1, C1, C2); and a growing confidence to express critical views about KWTRP (C1 vid4; and C2 vids 1&3).

With time, teachers also felt increasingly able to leave AD and NM to conduct follow-up meetings independently with students and frequently made comments such as “*the process is educative for the students and good for their language skills”* (Male, School LI1principal). Table 3 summarizes the ways in which AD, NM and participating students gained from the PV process.

**Table 3:** Summary of gains for the SEP and students from the PV process.

**Gains for the SEP**	**Gains for students**
An evaluative understanding of the influence of SEP	Learning about film-production and enjoyment of the process
Insights into the context of SEHR in Kilifi	Increased confidence in communicating with research staff
An appreciation of the depth of engagement required to facilitate learning of research concepts	Greater depth of understanding about research and KWTRP

**Figure 3:**
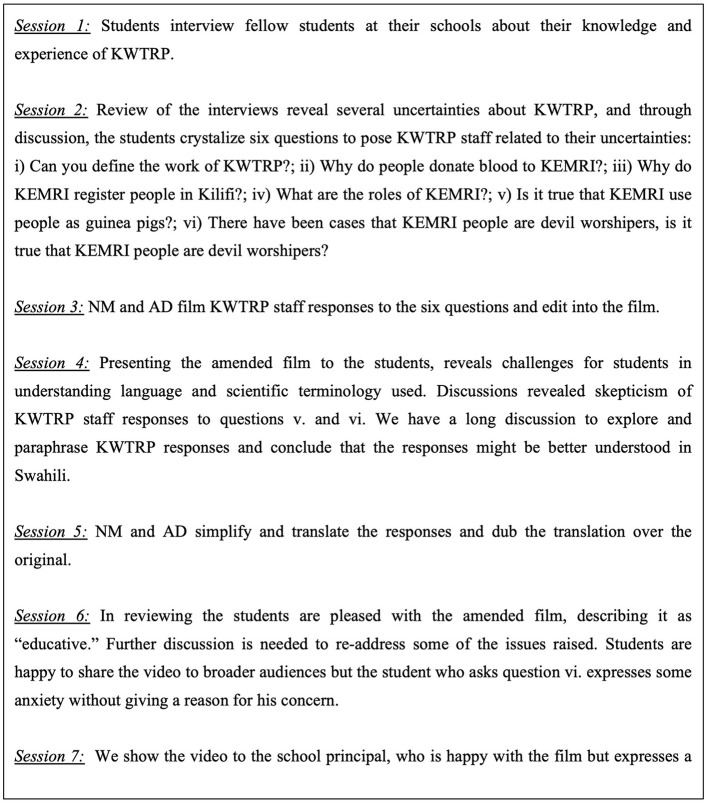
C1vid4 case study of further engagement facilitated by PV.

Figure 3 highlights an iterative example of knowledge co-production between AD, NM and the students during the development (or production) of the C1vid4 film. The process of knowledge co-production was facilitated through the extended engagement afforded by PV which enabled students to critique, question and learn about research. It illustrates that over the PV process, whilst students learned about health research and gained confidence in articulating their questions, NM and AD gained a thorough appreciation of the depths of engagement required to facilitate student's learning about complex research procedures.

It became apparent throughout the duration of the PV process with all four groups that combining PV with participant observation provided a means of documenting student understanding of research and knowledge gaps whilst facilitating learning about research.

In some cases, the PV process enabled an understanding of how minimal exposure to the SEP activities could contribute to confusion about the role and requirements for employment at the KWTRP in general. For example, in reviewing C1vid4 (see Figure 3) with the students, it became clear that students could not differentiate between the use of blood samples in research, compared to blood taken for the transfusion service. Several discussions on consecutive weeks were required to address this challenge. In a second example, students in schools FTF1, LI1, and C1 referenced the KWTRP School Leaver's Attachment Scheme (SLAS) either in their films or in review discussions. They all accurately described the requirement of a mean grade of B+ and above in the Kenya Certificate of Secondary Education (KCSE) exams to apply for the scheme, and expressed that the internship provided valuable career experience. However, students from LI1 and C1, with little or no exposure to the SEP, expressed the misconception that all staff were recruited to KWTRP generally on the basis of their getting a B+ in their KCSE secondary school education exams. In both schools, this led to lengthy and repeated discussions between AD, NN and students about the qualification requirements for the school leaver's attachment scheme, work at KWTRP as a field worker, and qualification requirements needed to become a doctor and a nurse. Following the discussion, a C1 student who had understood the range of qualifications required for different types of jobs attempted to convince his reluctant friend by reasoning: “*Do you think all workers need a B*+*? Even the toilet workers or cleaners? We have several types of workers there; the toilet cleaners don't need to get a B*+” (Male, School C1 participant observation notes). This belief is likely to have resulted from hearing about the School Leaver's Attachment Scheme through a range of community engagement efforts and concluding that the B+ and above applied to *all* employment at KWTRP. Another alternative interpretation depicted by LI1, C1 and C2 students comprised a belief that KWTRP would provide bursaries either for school or university fees. The PV approach afforded time and a space to discuss and attempt to address alternative interpretations of KWTRP encountered over the duration of the process. The amount of time taken, and reluctance (among some) to accept explanations, highlights that differences in interpretations of research often cannot be resolved through single meetings and require lengthy discussion.

As an appreciation of some of the conceptions held by students about research and barriers to engagement were gained, students developed their confidence in articulating their views and their ability to engage with AD and NM. Gains to students and the SEP are summarized in Table 3.

### What Valuable Insights Did PV Provide on the Context in Which SEHR Is Situated?

Importantly, PV afforded an opportunity to observe, experience and learn, first-hand, over a period of 6 weeks, about the context in which a joint project between researchers and students was conducted during extracurricular time. This provided valuable insights and considerations for SEPs in general. The first consideration is that, in the context of working in a school in Kilifi, engagement with a small group of students can elicit feelings of envy among non-participating students. This was evidenced in schools FTF1 and LI1 in two ways: (a) non-participants expressing jealousy for not being part of the PV group, and (b) students not included in the IAS expressing jealousy of those who were (FTF1 Visit1 notes AD; School LI1 vid 3). Jealousy, in the context of SEHR activity, was evidenced further in school LI1's filmed interview about KWTRP, where one of the students related his experience of IAS: “*many people felt happy and the people who ignored it, they felt jealousy.”* (School Male, LI1 vid 3).

The second consideration is the time required for the PV process. Over the duration of the PV project, it became apparent that other competing activities and issues influenced student's ability and desire to participate in PV activities. These concurrent activities comprised county sports competitions and trainings in preparation for these, continuous assessment tests and exams, county poem, recital and drama competitions, after-school clubs (science club, Red Cross club and Straight Talk HIV club), school trips (History trip), and absenteeism. All engagement with schools, including PV, needs to take student's other obligations and commitment into account when planning.

A third consideration for SEP activities is understanding the many challenges students face in their day-to-day lives which can create barriers to their aspirations. While this understanding may have been achieved through alternative qualitative approaches, PV enabled students to dramatize their challenges in pursuing education, providing the viewpoint of multiple characters. The challenges illustrated in their performances comprised poverty and lack of money for fees to pursue studies, peer pressure related to drugs, sex and devaluing education, gender related issues serving as a barrier to girls' education, and corrupt employers with unfair employment practices. Examples of these barriers to education and the achievement of aspirations are illustrated in Table 4.

**Table 4:** Barriers to pursuing education.

**Hinderances to education**	**Illustrative example**
**Conflicting attitudes to education:** A portrayal of positive student attitudes toward education and a need to promote the value of education to the community	“*What is education? Have you ever thought that education helps in life? Be aware that education is the key to success. Don't just sit there, go for it.” (Female, School LI1 vid1)*.
**Financial barriers to education:** lack of ability to pay fees giving rise to ‘drop-out’	“*School fees is the biggest challenge people face. You can go to school to read but be chased away, it discourages (Male, School C2 vid 7)*
**Specific hinderances to girls' pursuit of education:** school drop-out due to pregnancy (FTF1 Vid4); approaches from boys on the way to school for relations, transactional sex or both combined with peer pressure (FTF1&LI1 Vid1; and FTF1 vid4); and forced marriage for dowry (LI1 Vid5).	*Teacher: Sidi, you were very bright but now you are pregnant, so you will go home and take care of your pregnancy. [Teacher gives Sidi a note] you will take that to your parent* *Sidi: How much then?* *Lowela: Five hundred shillings* *Lowela [whispering]: Iddi loves you* *Narrator: Sidi agrees to be loved by Iddi so that she doesn't annoy her friend Lowela* *Father: I told you I don't want to her those words of yours. We should marry away our child so that we get dowry money*. *Mother: We will spend that money and it will get finished, my husband. This child should study, do you hear me?* *Father: No, I have said she should drop out. I am the man of this house! (School LI1 vid 5)*
**Drugs as a barrier to education** portrayed in two films (C1_vid3 and C2_vid8) as causing disruption to studies and to class activities.	*Both films depict intoxication in the classroom following smoking “Bhang” (marijuana) procured from dealers near to the school*.
**Corrupt employment practices** hampering the achievement of aspirations	“*I try whilst other cry” (C2 Vid6) depicts a job interview where the interviewer asks the candidate interviewees for a bribe: “scratch my back, I scratch yours.” The first applicant virtuously refuses to bribe whilst the second is rewarded with the promise of employment after agreeing “to use [his] pocket” and pay a bribe*.

## Discussion

The PV process undertaken with groups of students from four Kilifi secondary schools, provided some evaluative evidence of the influence of the SEP in promoting student understanding of research, confidence in articulating their understanding of KWTRP, aspirations toward medical and health related careers, and enjoyment in interacting with research staff. Perhaps unsurprisingly, this was most evident for students who interacted the most with the SEP. Whilst our use of PV, in comparison to traditional evaluation approaches, may be limited in terms of controlling for confounders and making generalizable claims about SEHR, it offered valuable insights into SEHR practice which could not have been made through surveys. Used as an evaluation tool alongside a pre and post survey with intervention and control groups, PV has corroborated impact data (6), but has also provided a greater depth of understanding of the context in which engagement operates and which can be drawn upon to inform future SEHR in Kenya.

A potential complicating factor, though not unique to this study, is the possibility of acquiescence bias (78), which might account for the absence of critical comments about the SEP by students. On one hand, it could be that SEP activities were novel and universally enjoyed by students, but on the other, it is important to consider that students may have avoided being critical of the activities to please NM and AD and to avoid jeopardizing perceived future benefits from KWTRP SEP. The initial shyness of some students may have been caused by limited exposure to KWTRP researchers, including white middle-aged men (AD), and/or a prevailing school/home culture of girls remaining quiet in public discussions where boys are present. Our observation notes document a growth in student confidence and rapport with NM and AD over the project's duration. This is likely to have strengthened the relationship and gradually nurtured the students' willingness to voice their opinions. We argue that the extended interaction is likely to have fostered a willingness among students to share honest opinions.

In addition to providing evaluative information about SEHR, more importantly PV proved to be a valuable engagement method in itself, where KWTRP researchers and students learned about each other. While it could be argued that a similar degree of “openness” may have been attainable if a comparable amount of contact time was spent in creating rapport with students prior to FGDs, PV offered an opportunity for the rapport to be nurtured over a creative “arts-based” collaboration between researchers and students. In a similar way to the IAS online engagement activity, the novelty of the PV approach and activities contributed to the students' overall motivation to participate. Ethnographers participate in the day-to-day lives of research participants over periods of time, to draw inferences based on observations and discussions (71). They describe “ecological validity” as a strength of ethnographic data emerging from observing natural everyday life, compared to data emerging from “experimental” conditions such as surveys and time-constrained FGDs. The PV method in the SEP evaluation, placed students in novel film-making situations, as opposed to observing day-to-day life events, and offered students an opportunity to learn about filmmaking and nurture their communication and confidence. Thus, in using PV as an ethnographic tool, for students unfamiliar with film-making, there is a potential trade-off between the loss of “ecological validity” of data emerging from observing participants in their “natural” environment, and PV's promise of enhancing communication through leveling power differences between researcher and researched (65). The PV may not have fully ameliorated differences between AD, NM and students in all cases, however, its use as an arts-based tool for knowledge co-production (59) afforded time where students nurtured the confidence to share questions, opinions, satisfaction and dissatisfaction, not only in relation to film-making, but also in relation to SEHR, KWTRP, research and their own aspirations. From the point of view of a SEP evaluation, spending time at the schools in a co-production project offered important *in-situ* insights into how a SEHR activity works in the context of day-to-day school life. Further, and perhaps most importantly, with the ability to prioritize, delete, re-shoot and select preferred scenes, over the duration of the PV project, students were able to refine the content they wished to articulate in their videos. This arguably points to the students' growing “ownership” of the film-making process through the experience of having a “stake in the idea(s)” shared, feeling that the ideas shared were relevant and having their ideas valued (79, 80). For the use of PV in evaluation, we feel that increased ownership nurtures participants' confidence in sharing views honestly, therefore contributing to the finding's validity and authenticity.

While SEHR activities, and the PV project may have provided benefits and enjoyment for most participants (Table 3), our study provides insights into contextual challenges faced by students in their already busy schedules for curricular and extracurricular activities. For the students, the SEP is comparable to a single book on a wide and crowded bookshelf of competing activities and circumstances. For many, the novelty of the SEP activities, including the PV project, and the opportunity for interaction with KWTRP researchers may have been inspirational and enjoyable, but for others it was another set of activities competing for space in their thoughts. This underscores a priority need for engagement practitioners to carefully plan activities to ensure that they maximize enjoyment and benefits for students and schools. Important to emphasize is that interpretations of “benefits” may differ between the standpoints of researchers, school teachers and students. For example, students and teachers may not necessarily consider an enhanced understanding of locally conducted health research as being a priority benefit. It is also important to recognize the limits of community engagement and related activities in addressing some of the structural challenges faced by students, often related to limited resources and poverty (1).

The PV approach used in this study is not without limitations as an evaluation tool. It requires a broad range of researcher skills, from facilitation, videography and editing, to participant observation and qualitative analysis. It is time- and resource-heavy in ensuring consent at several levels and different time points (44), and only captures the views of relatively few participants. However, in the interest of making SEHR, including its evaluation, beneficial and enjoyable for students, PV, unlike other research methods, presents a considerate way of drawing from student's time, through providing opportunities to gain personally from the experience.

In our experience, as well as other's (51, 65), PV led to AD, NM and students learning alongside each other. As students honed their communication skills, learned about film-making and gained a deeper understanding of research processes through discussion and subsequent amendment of their films, AD, NM and KWTRP engagement team were offered insights into student lives and an appreciation of the depth of engagement required to address alternative interpretations of research.

Enabling the students to decide on the content of the films related to achieving their education and career aspirations has opened a new understanding of the context within which the KWTRP's research takes place and the complexity of community members lives. Lavery et al. (81) describe “*build[ing] knowledge of the community, it's diversity and it's changing needs”* as an important consideration “*for effective community engagement.”* This PV process has contributed not only to an understanding about the SEP intervention, but also, and perhaps more importantly, has provided insights into the context in which SEHR takes place, and which in turn can influence participation in activities. This makes PV, in itself, a potentially strong tool for engagement and evaluating engagement.

### Conclusion

Our study contributes to the field of SEHR through highlighting the value of PV, not only as an evaluation tool, but also as a means of engaging school students further with health research. PV as an evaluation tool, yielded evidence of the SEP's influence on the students' views, attitudes, and aspirations. It also highlighted unintended consequences of SEP and a greater depth of understanding of the context in which SEHR takes place which can influence school and student participation. Our use of PV has illuminated the many struggles students face in pursuing their aspirations, and the important curricular and extra-curricular activities which compete against SEHR for students' time and attention. These insights compel us to ensure that engagement activities are enjoyable to students, beneficial from their point of view and mindful of their time and busy schedules. In addition to facilitating evaluation, PV was a valuable method of engaging students with health research, enabling researchers and students to learn alongside each other. Given the constraints on student and researcher time, methods which enable concurrent engagement and evaluation, conferring benefits to both researchers and students, should be embraced.

## Data Availability Statement

The datasets presented in this study can be found in online repositories. The names of the repository/repositories and accession number(s) can be found at: https://doi.org/10.7910/DVN/VP5K7I.

## Ethics Statement

The studies involving human participants were reviewed and approved by Scientific and Ethical Review Unit, Kenya Medical Research Institute, Kenya. Written informed consent to participate in this study was provided by the participants' legal guardian/next of kin.

## Author Contributions

The study was part of AD's Ph.D. project, supervised by CH, RH, and CJ, with assistance in carrying out the fieldwork from NM. CH and NM provided additional methodological support, but the study design and execution was the responsibility of AD. All authors contributed to manuscript revision, read, and approved the submitted version.

## Funding

This study was supported by Wellcome Trust International Engagement Award 100602/Z/12/Z.

## Conflict of Interest

AD, NM, and CJ were employee by The KEMRI-Wellcome Trust Research Programme. The remaining authors declare that the research was conducted in the absence of any commercial or financial relationships that could be construed as a potential conflict of interest.

## Publisher's Note

All claims expressed in this article are solely those of the authors and do not necessarily represent those of their affiliated organizations, or those of the publisher, the editors and the reviewers. Any product that may be evaluated in this article, or claim that may be made by its manufacturer, is not guaranteed or endorsed by the publisher.
